# Low-density lipoprotein cholesterol drives multinucleated giant cell formation in response to *mycobacterium bovis* Bacille Calmette-Guérin

**DOI:** 10.1038/s41598-025-34088-y

**Published:** 2026-01-07

**Authors:** Philipp-Alexander Merck, Anne Kathrin Lösslein, Jana Neuber, Stephan Schwer, Wenke Jonas, John Andrew Pospisilik, Ingo Hilgendorf, Philipp Henneke, Florens Lohrmann

**Affiliations:** 1 Medical Faculty, Institute for Infection Prevention and Control, University Medical Center, University of Freiburg, Freiburg, Germany; 2 Medical Faculty, Institute of Microbiology and Hygiene, University Medical Center, University of Freiburg, Freiburg, Germany; 3 Faculty of Biology, University of Freiburg, Freiburg, Germany; 4 Department of Experimental Diabetology, German Institute of Human Nutrition, Potsdam-Rehbrücke, Germany; 5 Max Planck Institute of Immunobiology and Epigenetics, Freiburg, Germany; and Department of Epigenetics, Van Andel Institute, Grand Rapids, USA; 6 Department of Cardiology and Angiology, Faculty of Medicine, University Heart Center Freiburg-Bad Krozingen, University of Freiburg, Freiburg, Germany; 7 Center for Chronic Immunodeficiency, Medical Faculty, University Medical Center, University of Freiburg, Freiburg, Germany; 8 Department for Pediatrics and Adolescent Medicine, Medical Faculty, University Medical Center, University of Freiburg, Freiburg, Germany

**Keywords:** Biochemistry, Diseases, Immunology, Microbiology

## Abstract

Macrophages play a central role in tuberculosis, both in bacterial persistence and tissue pathology. Body weight and a dysregulated lipid metabolism profoundly affect disease progression and survival. Yet, the relationship between lipid metabolism and mycobacterial immunity is still poorly understood. Accordingly, this study investigated the influence of cholesterol and lipoproteins on macrophage responses to mycobacteria, in particular the formation of multinucleated giant cells (MGC), which are key components of granulomas and involved in the containment of mycobacteria. We found that low-density lipoprotein (LDL) cholesterol was essential for the transformation of macrophage progenitors into MGCs in vitro, independent of the oxidation status. In contrast to these direct lipid effects on macrophage transformation, a lipid-rich diet to mice, which is known to elevate cholesterol levels in the blood, did not prime the MGC forming potential of bone-marrow progenitor cells. In conclusion, LDL promotes the mycobacteria-specific macrophage transformation, however, this effect appears to depend on tissue lipid availability rather than priming in the bone marrow.

## Introduction

Tuberculosis (TB), caused by *Mycobacterium tuberculosis* (Mtb), remains a major global health burden, particularly in low- and middle-income countries^1^. Most infections remain latent, but 5–10% of cases progress to active disease, often triggered by immunosuppression or malnutrition^2^. TB begins with the phagocytosis of Mtb by alveolar macrophages. In most cases, the immune system reacts by forming granulomas—immune cell aggregates that spatially confine the bacteria. Nonetheless, Mtb can also exploit granulomas for its persistence, replication and even spread^3,4^ and use lipid-rich macrophages as a survival niche^5^.

Malnutrition significantly impairs TB immunity, while moderate obesity may offer protective effects^6–8^, potentially mediated by increased levels of inflammatory cytokines^9^. However, dysregulated lipid metabolism in macrophages can promote Mtb survival potentially by impairing phagosome maturation and providing a nutrient source for bacteria^10^. Low-density lipoprotein (LDL) and high-density lipoprotein (HDL) are lipoprotein subclasses that transport cholesterol in the circulation. Oxidized LDL (oxLDL) represents a modified form of LDL resulting from oxidative stress, which promotes endothelial dysfunction, inflammation, and the progression of atherosclerotic lesions^11^. Emerging evidence suggests that dietary lipids and lipoproteins, including LDL and oxLDL, influence macrophage function and TB outcomes^12–14^. In addition, pharmacological modifications of lipid metabolism, e.g. via statins, can influence the TB disease course^15,16^.

Macrophages, the primary immune cells that intersect TB pathogenesis, adapt to the tissue environment at the site of infection and are the main components of TB-typical multicellular tissue structures called granulomas. Specialized macrophage types in mycobacterial granulomas are epithelioid cells, foam cells, a lipid-laden macrophage type, and multinucleated giant cells (MGC)^17^. Our previous in-depth characterization of MGCs showed that they develop from myeloid bone marrow progenitors by modified cell division, integrating TLR2-dependent signals with DNA damage sensing. Furthermore, MGC exhibited altered lipid metabolism, including an accumulation of cholesterol, which may enhance bacterial survival^5,18,19^.

This study aimed to investigate the interplay between lipoproteins and the macrophage responses to mycobacteria, with a focus on MGC formation.

Methods

### Animals

Bone marrow was extracted from male adult C57BL/6 mice. Mice were handled according to local ethical standards and regulations concerning animal welfare. The reporting is in accordance with the ARRIVE guidelines. Mice used for this study without prior treatment were bred by the Center for Experimental Models and Transgenic Service (CEMT), University Medical Center and Faculty of Medicine, University of Freiburg. All animal experimentation protocols were approved by the Freiburg Regional Council – Department for Animal Welfare and Food Safety. The respective animal experiment permit numbers were G-19/180 and X-16/02F. All procedures were carried out in accordance with applicable institutional and governmental guidelines and regulations.

Mice fed high fat diet (HFD) for the indicated duration as well as age-adjusted normal caloric (NC) control mice were bred at three different institutions. 1) the Center for Experimental Models and Transgenic Service (CEMT), University Medical Center and Faculty of Medicine, University of Freiburg (Ingo Hilgendorf), 2) the Max Planck Institute of Immunobiology and Epigenetics, Freiburg (Andrew Pospisilik), or 3) the Department of Experimental Diabetology, German Institute of Human Nutrition, Potsdam-Rehbrücke (Wenke Jonas). Different commercial chows were used: Research Diets D12451 (45 kcal% from fat, 35 kcal% from carbohydrates, 20 kcal% from proteins) for mice from 1) and 3). Or Research Diets D12492 (60 kcal% from fat, 20 kcal% from carbohydrates, 20 kcal% from proteins) for mice from 2). The duration of treatment was 6-7 months for high-fat diet versus normal diet group, here, mice treated in locations 1) and 2) are combined for the MGC quantification assay. For the qPCR analysis, mice from location 1 were used, they were fed for 3 months either HFD or NC. In location 3) mice were treated for one month with high-fat diet ad libitum versus high-fat diet in an intermittent fasting scheme. Group sizes were 4-5 in the HFD/NC group and 7-8 in the intermittent fasting group. Animals were weighed before analysis to confirm efficacious dietary interventions. For this study, only the bone marrow was analyzed or cultured further.

Mice were sacrificed by exposure to carbon dioxide (CO₂) in accordance with institutional guidelines as well as AVMA guidelines^20^. Mice were euthanized by CO₂ inhalation using a displacement chamber. Animals were placed in their home cage (or a dedicated transparent chamber) and exposed to carbon dioxide (CO₂) delivered from a compressed-gas cylinder via a pressure-reducing regulator and flow meter. The flow was set to ensure a CO₂ concentration increase corresponding to 30–70% chamber volume replacement per minute, until respiratory arrest was observed. After the last animal ceased breathing, CO₂ flow was maintained for an additional 1 minute to ensure death. Death was confirmed by absence of respiration and heartbeat, and then cervical dislocation was performed to assure death. Carcasses were then removed and processed for downstream analysis. All personnel performing euthanasia were trained and certified according to local regulations.

### Bacteria

Throughout the study, the strain *Mycobacterium bovis* BCG-medac/Vejicur (Bacillus Calmette-Guérin) was used. Live *Mycobacterium bovis* Bacillus Calmette-Guérin (BCG)^21,22^ were kindly donated by Prof. Dr. Dirk Wagner (Division of Infectious Diseases, Department of Internal Medicine II, University Medical Center and Faculty of Medicine, University of Freiburg). Live *Mycobacterium bovis* Bacillus Calmette-Guérin were stored as aliquots of 10^8^ pathogens at -80°C until usage. After thawing and resuspension, the suspension was sonicated in a stirred water bath for 30 seconds and subsequently vortexed vigorously. These two steps were performed three times. Thereafter, the bacteria were heat-fixed by placing them in a heated shaker at 80°C, 600 rpm for 30 minutes. All stimulation experiments were performed with heat-fixed bacteria.

### Isolation of murine bone marrow cells and cultivation of bone marrow derived macrophages (BMDM)

Bone marrow was flushed out of long bones through a 70 µm strainer and cultured in DMEM containing 10% heat-inactivated FBS (PAN-Biotech) and 5 µg/ml of ciprofloxacin and 20 ng/ml of macrophage colony-stimulating factor (M-CSF) in an incubator at 37°C, 5% CO_2_ for five days. Then, adherent cells were harvested using a scraper.

### Fluorescence activated cell sorting (FACS) of myeloid progenitors

Magnetic activated cell sorting (MACS) of murine bone marrow cells was performed to enrich for progenitors. Following cell counting, bone marrow cell suspensions were diluted with FACS buffer (PBS with 2% FBS and 2 mM EDTA) and biotin antibodies (see table 1) to a total volume of 100 µl per 10^7^ cells and incubated for 20 minutes at 4°C.

**Table 1:** Biotin antibodies used for MACS

**specificity**	**antibody type**	**label**	**dilution**	**manufacturer**
CD3ε	hamster IgG1κ	Biotin	1:100	MiltenyiBiotech
CD19	rat IgG2aκ	Biotin	1:100	MiltenyiBiotech
Siglec F	rat IgG1	biotin	1:100	MiltenyiBiotech
Ter119	rat IgG2bκ	biotin	1:100	MiltenyiBiotech
Ly6G	rat IgG2aκ	biotin	1:100	BioLegend
CD127	recombinant human IgG1	biotin	1:100	MiltenyiBiotech
Sca-1	rat IgG2aκ	biotin	1:100	MiltenyiBiotech

Cells were incubated with the indicated antibodies for 20 minutes. After washing, negative selection was performed using Dynabead® solution (30 µl/10^7^ cells) and magnetic beads according to the manufacturers’ instructions. Then, cells were stained (see table 2) at 4°C in 100 µl of FACS buffer per 10^7^ cells for 30 minutes, protected from light.

**Table 2:** Fluorescent antibodies used for flow cytometry and FACS

**specificity**	**antibody type**	**label**	**dilution**	**manufacturer**
CD11b	rat IgG2bκ	APC-e780	1:2000	eBioscience, Thermo Fisher Scientific
CD115	rat IgG2aκ	PE	1:20	MiltenyiBiotec
CD117	rat IgG2b, κ	BV421	1:100	BioLegend
Ly6C	ratIgG2c, κ	PerCP-Cy5.5	1:400	eBioscience, Thermo Fisher Scientific
CD115-isotype	rat IgG2aκ	PE	1:135	eBioscience, Thermo Fisher Scientific

For sorting, we defined cMoP (common monocyte progenitors) as CD115^+^, CD117^+^, CD11b^-^.

### Generation of multinucleated giant cells (MGC) from BMDM or myeloid progenitors

MGC cultivation medium was prepared using Opti-MEM™+GlutaMAX™ containing 10% heat-inactivated FBS (PAN-Biotech) and 5 µg/ml of ciprofloxacin.

BMDM or sorted myeloid progenitor populations (MDP, cMoP, iMoP and MC) were suspended in MGC cultivation medium. They were then seeded into 96-well plates (CellBIND®, Corning) at 5,000 cells per well. For subsequent examination of the supernatant cells were seeded into 35 mm culture dishes (CellBIND®, Corning) at 60,000 cells per dish.

All cells were stimulated with 50 ng/ml of M-CSF alone or 50 ng/ml of M-CSF plus heat-fixed *Mycobacterium bovis* Bacillus Calmette-Guérin (BCG, 10^5^ or 10^6^ per ml) and incubated at 37°C, 5% CO_2_ for one, two, four, six or eight days. Of note, no experiments with live bacteria were performed in this study. If indicated, human lipoproteins (HDL, LDL, oxLDL, Athens Research & Technology) were added to the cell culture medium. After the stimulation, all stimulants, including the heat-fixed bacteria were left on the cell culture for the whole incubation period, no medium exchange was performed.

### MGC quantification

MGCs were manually quantified using Hemacolor® rapid staining according to manufacturers’ instructions and image acquisition by bright field microscopy (Axio Observer.Z1/7 by Zeiss, 10x objective) and ZEN software (black edition, Carl Zeiss AG).

Each well of the used 96-well plates (CellBIND®, Corning) has a cell growth area of approximately 32 mm^2^. To reduce bias, nine representative images were taken from each well, always using the same positions within the wells. The nine images covered 5.22 mm^2^ per well (corr. approx. to 16% of total growth area). Cell numbers were quantified manually using ZEN software (blue edition, Carl Zeiss AG). MGC were defined as cells containing three or more nuclei and having a bigger cell area than average BMDM stimulated with M-CSF.

### Quantification of lipids

Samples of MGC culture supernatant were sent to the Department of Lipid Analytics of the Institute of Clinical Chemistry and Laboratory Medicine, University Medical Center and Faculty of Medicine, University of Freiburg, where their lipid content was examined via ultracentrifugation.

### Delipidation of FBS

Fumed silica powder was added to FBS (PAN-Biotech) at a concentration of 20 mg/ml. The suspension was placed on a rocking table for three hours at 4°C. After centrifugation at 2000g for 15 minutes, the supernatant consisting of the delipidated FBS was stored at 4°C in the dark.

### qPCR

For cell lysis and RNA isolation, the RNeasy@ Plus Micro Kit (Qiagen) was used according to manufacturer’s instructions. Lysates were stored at -80 °C until used for RNA isolation. Isolated RNA was subsequently transcribed into complementary DNA (cDNA) using the SuperScript™ IV VILO™ Master Mix (Invitrogen, Thermo Fisher Scientific) for concentrations of 10ng/µl RNA or below or the iScript™ cDNA Synthesis Kit (Bio-Rad Laboratories, Inc.) for samples with a concentration of >10 ng/µl RNA, both according to manufacturer’s instructions.

cDNA was stored at -20°, qPCR was performed using SYBR™ Green PCR Master Mix (Thermo Fisher Scientific) and specific primer sequences for each gene (Sigma-Aldrich, see below). qPCR was conducted using a real time thermocycler (mastercycler realplex EP gradient S, Eppendorf) and its software.

Threshold cycles (ct) were measured for each gene and normalized relative to the expression of glyceraldehyde 3-phosphate dehydrogenase (*Gapdh*). This Δct value was obtained by subtracting the mean ct value of the triplicate samples of GAPDH from the mean ct value of the triplicates of the gene of interest, see table 3 for primer sequences. mRNA expression levels were depicted as 2^(-Δct).

**Table 3:** Primer sequences

**gene**	**sequence forward 5’-3’**	**sequence reverse 5’-3’**
*Abcg1*	CCT TCC TCA GCA TCA TGC G	CCG ATC CCA ATG TGC GA
*Apoe*	AAC CGC TTC TGG GAT TAC CT	TGT GTG ACT TGG GAG CTC TG
*Gapdh*	ACT CCA CTC ACG GCA AAT TC	TCT CCA TGG TGG TGA AGA CA
*Nos2*	ACC CTA AGA GTC ACC AAA ATG GC	TTG ATC CTC ACA TAC TGT GGA CG
*Il1b*	TTC AGG CAG GCA GTA TCA CTC	GAA GGT CCA CGG GAA AGA CAC

### Statistical analysis

Statistical analysis was conducted using GraphPad Prism v10 software. All data are shown as arithmetic means ± standard deviation (SD). All plots depict data from at least three independent experiments. Fig. 2A-B contains data from 2 independent measurements; Fig. 2C contains data from 3 independent measurements. Fig. 5 contains data from at least 4 mice per condition from 2 independent experiments.

**Fig. 1:**
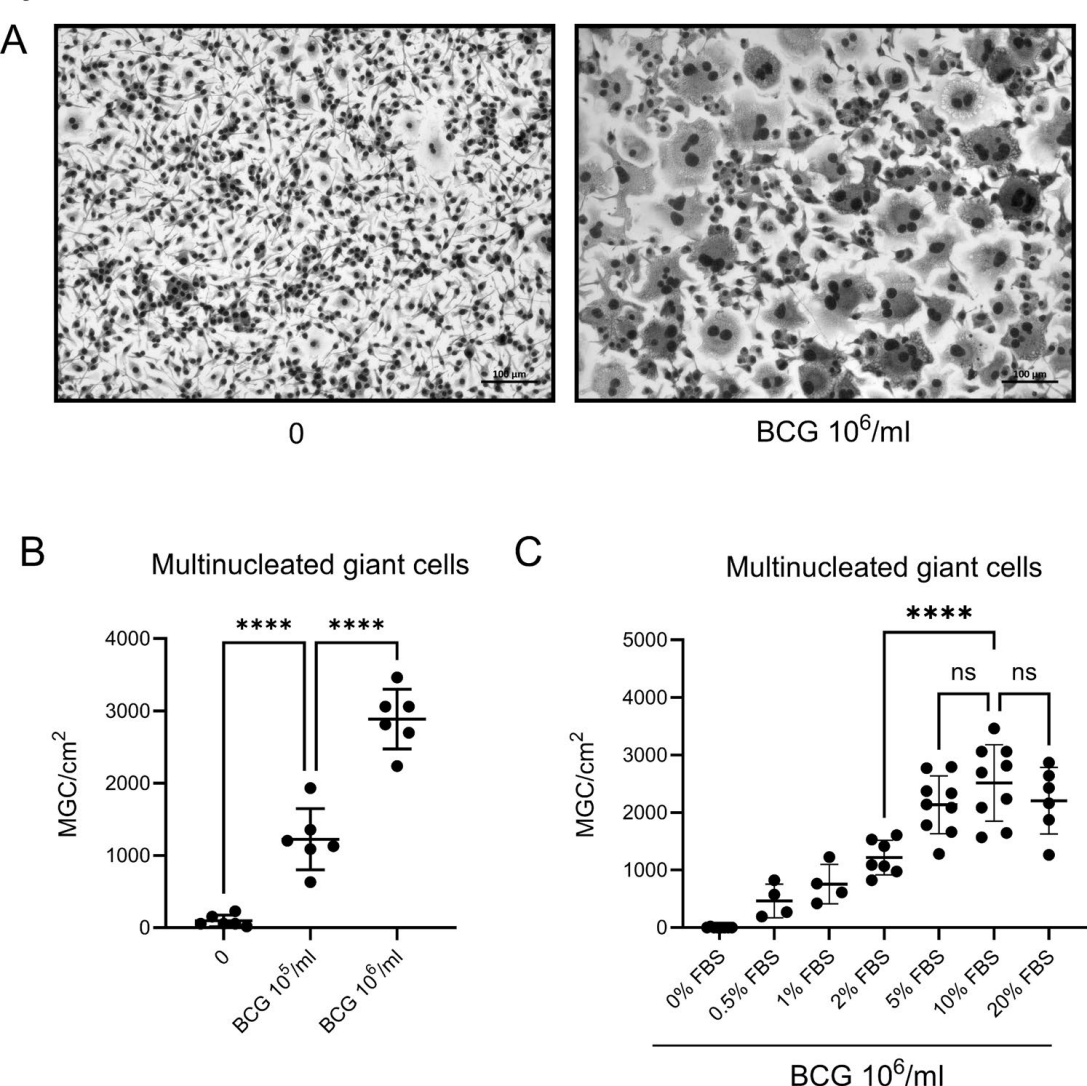
**A** Exemplary bright-field microscopy pictures of BMDM, left unstimulated or treated with the indicated dose of heat-fixed BCG for 6 days. Scale bar 100 µm. **B**: Quantification of N° of MGC per cm^2^ of culture plate after 6 days of culture stimulated with indicated dose of heat-fixed BCG. One-way ANOVA. **C**: Quantification of N° of MGC per cm^2^ of culture plate after 6 days of culture stimulated with 10^6^ particles heat-fixed BCG/ml and increasing concentration of FBS. Reference for statistical testing: 10% FBS. Significance for exemplary meaningful comparisons is displayed. One-way ANOVA. BMDM: Bone marrow-derived macrophages; BCG: Bacille Calmette-Guérin; MGC: Multinucleated giant cell. FBS: Fetal bovine serum.

**Fig. 2:**
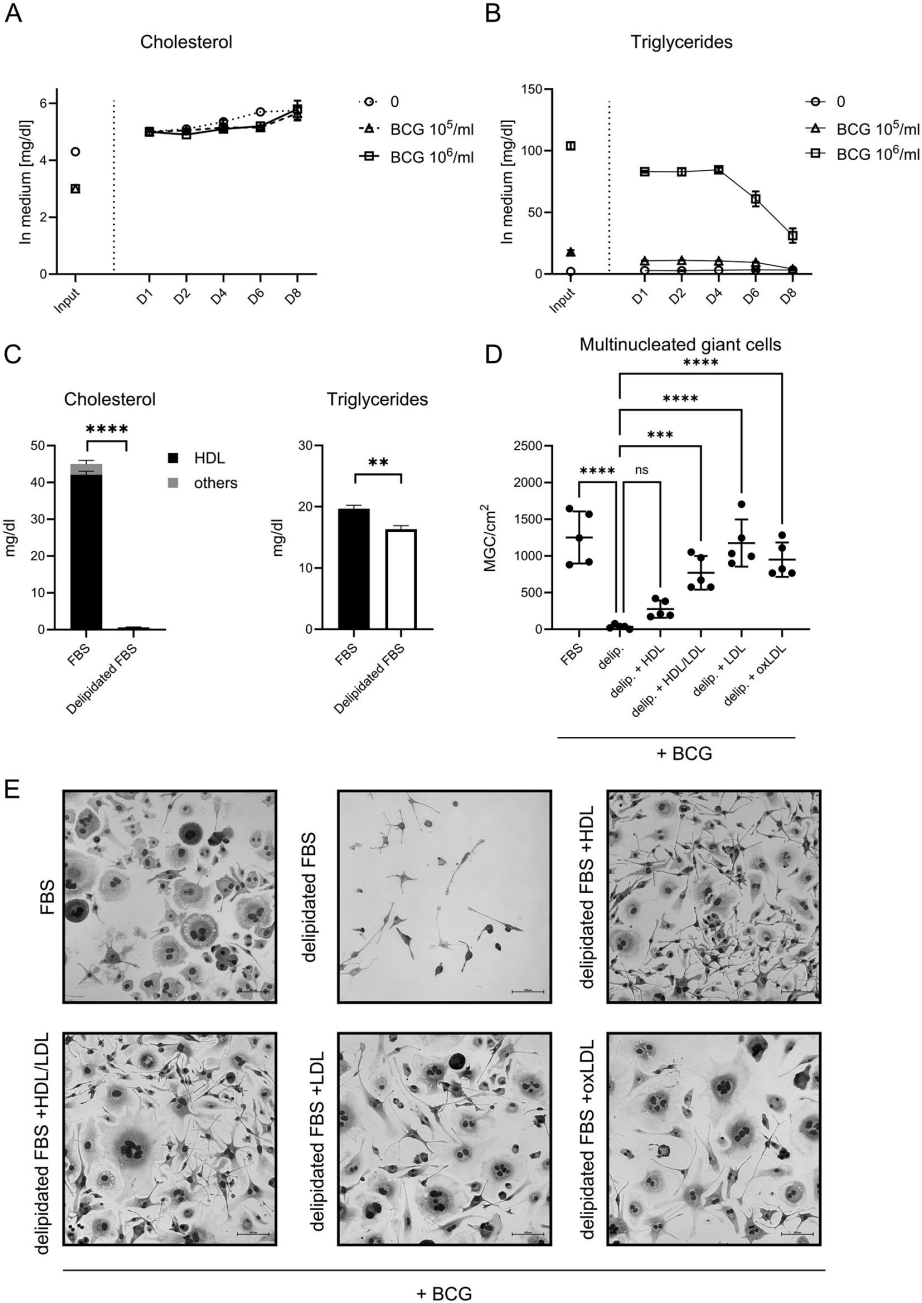
**A**, **B**: Total cholesterol (**A**) and total triglyceride (**B**) content in input medium and culture supernatant during MGC transformation over a time course of 8 days (two independent measurements). **C**: Total cholesterol (split into HDL and others) and total triglyceride content in untreated FBS or FBS after fumed silica delipidation (three measurements). t-test. **D**: Quantification of N° of MGC per cm^2^ of culture plate after 6 days of culture stimulated with 10^6^ particles heat-fixed BCG/ml. Supplemented either with 10% FBS, 10% delipidated FBS or 10 % delipidated FBS + indicated lipoprotein (containing total amount of cholesterol equal to 10 % regular FBS). Reference for statistical testing: Delipidated FBS. One-way ANOVA. **E**: Exemplary bright-field microscopy pictures of BMDM for each condition displayed in D. Scale bar 100µm. BMDM: Bone marrow-derived macrophages; BCG: Bacille Calmette-Guérin; MGC: Multinucleated giant cell; FBS: Fetal bovine serum; HDL: High-density lipoprotein; LDL: Low-density lipoprotein; oxLDL: oxidized low-density lipoprotein.

**Fig. 3:**
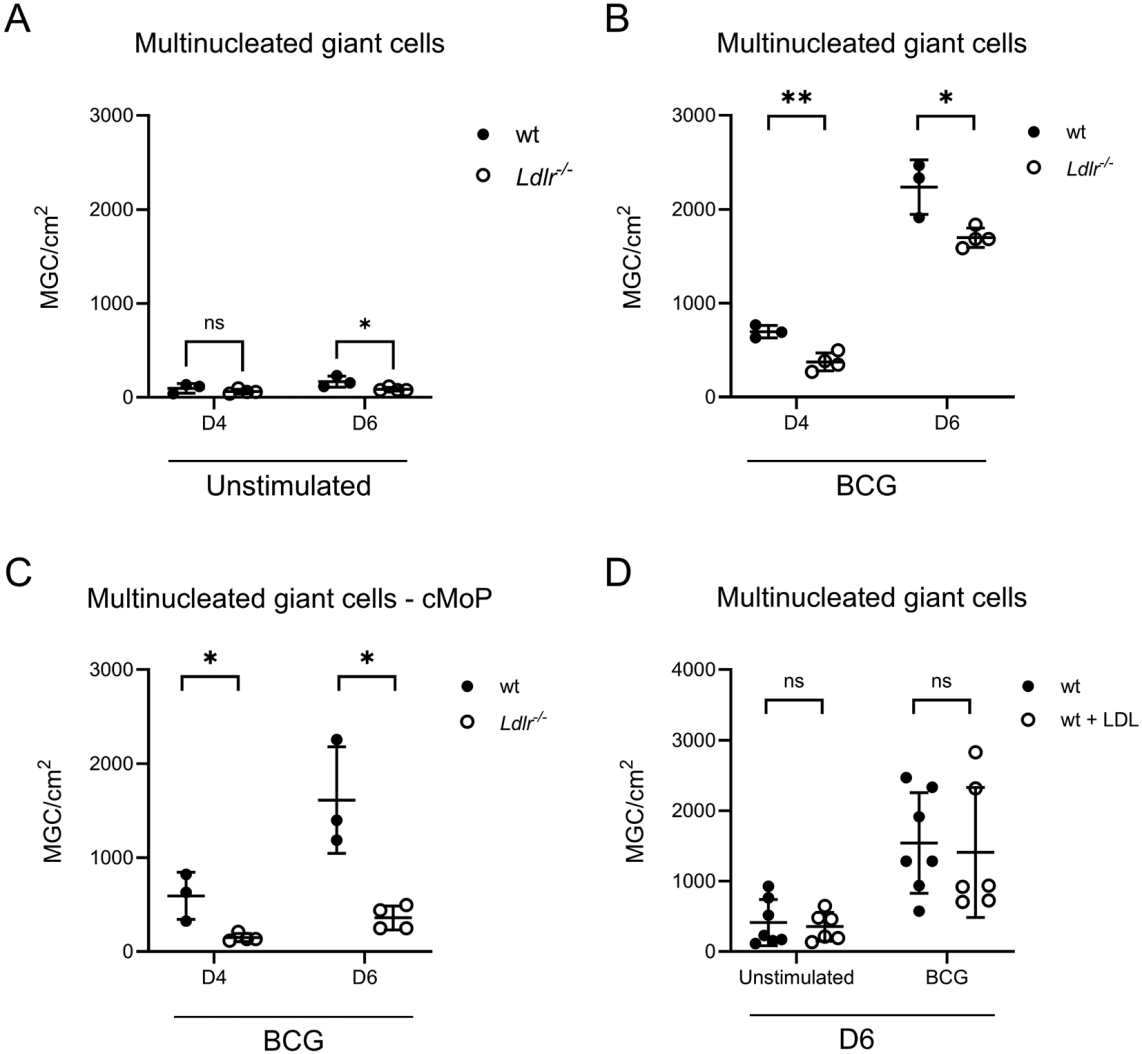
**A** Quantification of N° of MGC per cm^2^ of culture plate after indicated time of culture without stimulation. BMDM from either wt or *Ldlr*^*-/-*^ mice. Serial t-test. **B**: Quantification of N° of MGC per cm^2^ of culture plate after indicated time of culture stimulated with 10^6^ particles heat-fixed BCG/ml. BMDM from either wt or *Ldlr*^*-/-*^ mice. Serial t-test. **C**: Quantification of N° of MGC per cm^2^ of culture plate after indicated time of culture stimulated with 10^6^ particles heat-fixed BCG/ml. cMoP from either wt or *Ldlr*^*-/-*^ mice. Serial t-test. **D**: Quantification of N° of MGC per cm^2^ of culture plate after indicated time of culture stimulated with 10^6^ particles heat-fixed BCG/ml and supplemented with or without 50 µg/ml LDL. BMDM from wt mice. Serial t-test. BMDM: Bone marrow-derived macrophages; BCG: Bacille Calmette-Guérin; MGC: Multinucleated giant cell; FBS: Fetal bovine serum; LDL: Low-density lipoprotein. cMoP: common monocyte progenitor.

**Fig. 4:**
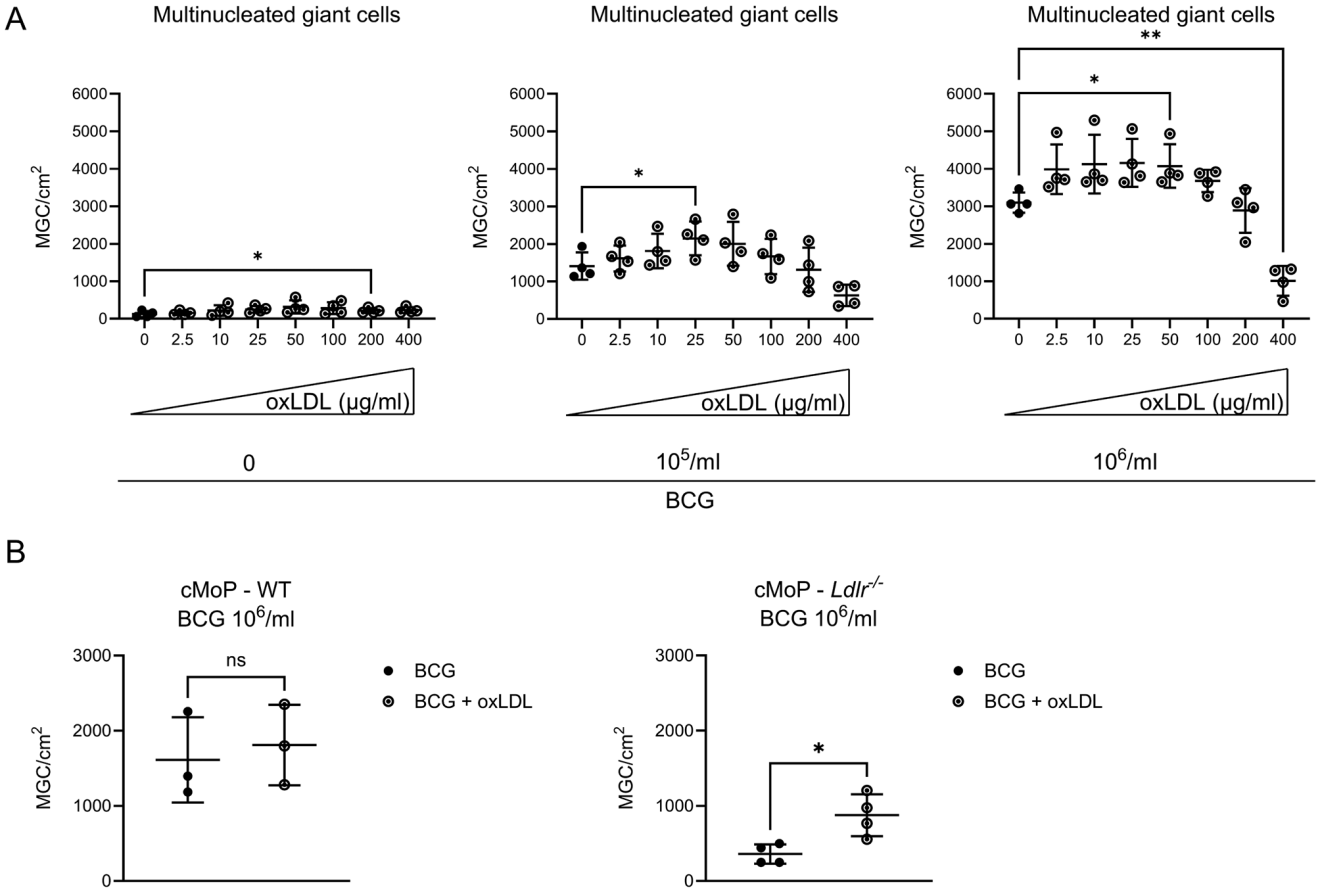
**A**: Quantification of N° of MGC per cm^2^ of culture plate after 6 days of culture, without stimulation (left), or stimulated with 10^5^ (middle) or 10^6^ (right) particles heat-fixed BCG/ml. Supplemented with 10% FBS alone or 10% FBS + increasing concentrations of oxLDL (indicated as concentration of lipoprotein-associated cholesterol in µg/ml). Reference condition for statistical testing was the condition without oxLDL in each of the three analyses. All significant comparisons are indicated. One-way ANOVA. **B**: Quantification of N° of MGC per cm^2^ of culture plate after indicated time of culture stimulated with 10^6^ particles heat-fixed BCG/ml and supplemented with or without 50 µg/ml oxLDL. cMoP from either wt (left) or *Ldlr*^*-/-*^ (right) mice. t-test. BCG: Bacille Calmette-Guérin; MGC: Multinucleated giant cell; FBS: Fetal bovine serum; oxLDL: oxidized low-density lipoprotein.

Whenever two distinct groups were compared, the unpaired t-test was used. Multiple t tests were corrected with using the Holm-Šídák method (fig. 2C, 3, 5B) . With more than two groups, one-way analysis of variance (ANOVA) was performed (fig. 1B, 5A). If multiple conditions were compared to one reference condition, this was followed by Dunnet’s multiple comparisons test (fig. 1C, 2D, 4).

P values of < 0.05 were defined as statistically significant. In graphs, asterisks were used to represent significance as follows:

P value ≥ 0.05 ns, < 0.05 *, < 0.01 **, < 0.001 ***, < 0.0001 ****

### Software

Table 4 lists the versions of the softwares used for this study.

**Table 4:** Analysis software used

**program**	**manufacturer/ license**
GraphPad Prism 10.2	GraphPad Software, LLC
Kaluza Analysis 2.1	Beckman Coulter GmbH
Kaluza for Gallios, acquisition software G1.0	Beckman Coulter GmbH
ZEN 2.6 (blue edition)	Carl Zeiss AG
ZEN 2.3 (black edition)	Carl Zeiss AG

## Results

Stimulation of monocyte progenitor cells, or bone marrow-derived macrophages (BMDM), with heat-fixed *Mycobacterium bovis* Bacillus Calmette-Guérin (BCG) induces differentiation into a multinucleated giant cell (MGC) morphology in a subset of macrophages in a dose-dependent manner (fig. 1A & B). Fetal bovine serum (FBS) was essential to MGC differentiation, and its effects are dose-dependent, with maximal stimulation achieved at 5 to 10% (fig. 1C).

Next to a cocktail of growth hormones, FBS contains high levels of cholesterol, which remain stable during eight days of MGC differentiation without media change (fig. 2A). Triglycerides were equally present, and levels were increased by adding heat-fixed BCG, which is known to contain high levels of triglycerides in the bacterial cytosol^23^. These levels slowly decreased after 4 days of cultivation (fig. 2B). Of note, 90% of cholesterol in FBS was found in the form of HDL in FBS (fig. 2C, left). To explore the impact of different lipids within the FBS, we delipidated it with fumed silica. This efficiently removed cholesterol from FBS^24^ while only mildly reducing triglycerides (fig. 2C). Supplementing media with delipidated FBS completely abrogated MGC formation in response to BCG. Addition of low-density lipoprotein (LDL) cholesterol to delipidated FBS fully recovered induced MGC formation. In contrast, high-density lipoprotein (HDL) cholesterol did not restore MGC formation (fig. 2D & E). Next, we asked whether the observed effects specifically resulted from engagement of the LDL receptor. However, LDL, which binds to specific LDL receptors, and oxidized LDL (oxLDL), which binds to scavenger receptors, did not differ in MGC induction with respect to number of induced MGC (fig. 2D) or their morphology (fig. 2E), suggesting therefore that LDL cholesterol itself, rather than activation of the receptor, is critical for MGC differentiation.

Next, we analyzed BMDMs from LDL-R knockout mice (*Ldlr*^*-/-*^) and found them to induce significantly less MGCs in culture, when left unstimulated (fig. 3A), and when stimulated with BCG (fig. 3B), confirming a non-redundant role for LDL in the transformation. We have previously shown that specific monocyte progenitor types in the bone marrow differ in their capacity to transform into MGC when stimulated with BCG or TLR2 ligands^19^. We therefore assessed the capacity of common monocyte progenitors (cMoP), the most potent MGC progenitor, of wt and *Ldlr*^*-/-*^ mice to undergo BCG-stimulated transformation to MGCs. We found that abrogating LDL signaling significantly reduced MGC formation (fig 3C). To complement these findings, adding LDL on top of FBS to unstimulated cells did not suffice to further induce MGC (fig. 3D). Similarly, adding LDL on top of FBS to BCG-stimulated cells did not further increase MGC formation (fig. 3D), in accordance with a saturation effect occurring already at the low levels of LDL present in FBS (fig. 1C).

Supplementing oxLDL, in addition to providing the full amount of LDL cholesterol-containing FBS, further increased the MGC forming capacity in a dose-dependent manner. Spontaneous MGC formation in the absence of BCG was modestly enhanced by oxLDL (fig. 4A, left), as was BCG-induced MGC formation in different stimulant doses (2.5 – 400 µg/ml of lipoprotein-associated cholesterol, fig. 4B, C). Beyond 50 µg/ml of oxLDL-cholesterol in the culture medium, MGC formation decreased along with observed morphological effects of general cellular toxicity in all conditions. To dissect relative contributions, we next stimulated wt and *Ldlr*^*-/-*^ cMoPs with BCG, and with or without adding 50 µg/ml oxLDL. Addition of oxLDL partially rescued the reduced MGC numbers on the *Ldlr*^*-/-*^ condition (fig. 4B). Thus, oxLDL facilitates MGC formation in a non-redundant manner together with LDL.

**Fig. 5:**
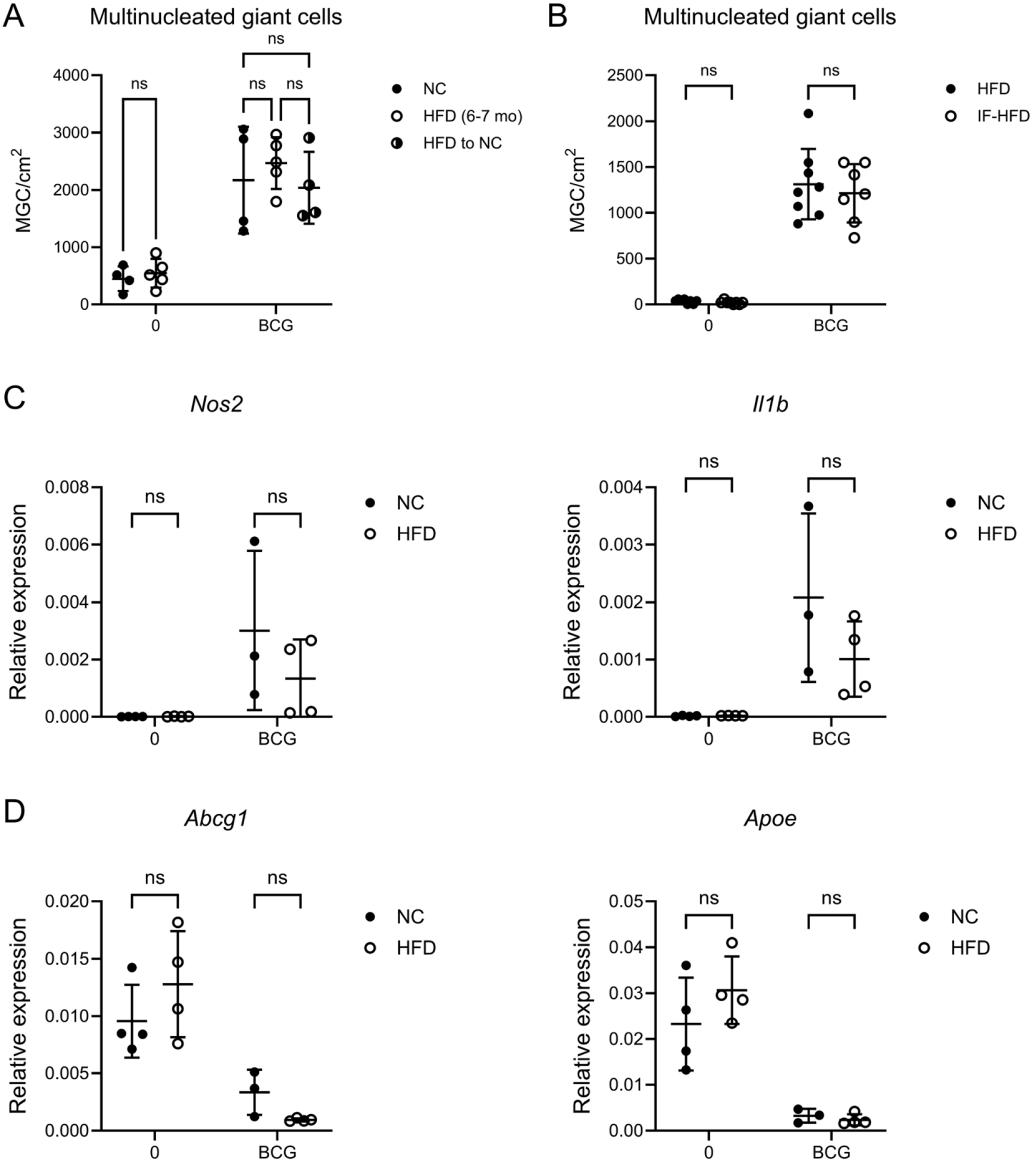
**A**-**B**: Quantification of N° of MGC per cm^2^ of culture plate after 6 days of culture, without stimulation, or stimulated with 10^6^ particles heat-fixed BCG/ml. BMDM from mice fed either **A**: Normal chow for 6-7 months (n = 4; mean weight at analysis 33.9 g +/- 2.3 g), or high-fat diet for 6-7 months (n = 5; 55.1 g +/- 6.6 g), or high-fat diet for 5-6 months followed by 1 month of normal chow (n = 4; 38.3 g +/- 2.7 g). **B**: High-fat diet for 1 month (n = 8; 25.3 g +/- 2.5 g) or subjected to intermittent fasting, i.e. cycling through 12h high-fat diet and 12h fasting for 1 month (n = 8; 22.8 g +/- 0.9 g). Two-way ANOVA. **C**-**D**: qPCR on cell culture lysates of cMoP after 6 days of culture, without stimulation, or stimulated with 10^6^ particles heat-fixed BCG/ml. Relative expression levels displayed as 2^(-ΔCt) compared to *Gapdh* levels. cMoP from mice fed either normal chow for 3 months (n = 4; mean weight at analysis 30.2 g +/- 1.7 g), or high-fat diet for 3 months (n = 4; 34.9 g +/- 3.5 g). Serial t-test with SH correction. BCG: Bacille Calmette-Guérin; NC: Normal chow. HFD: High-fat diet. IF: Intermittent fasting.

The nutritional status of the host influences susceptibility to tuberculosis^6^. At the same time, obesity directly and lastingly affects hematopoiesis^25^. Thus, we tested if high-fat diet (HFD) or relative fasting models changed the capacity of bone marrow progenitors to differentiate into MGCs. First, we used BMDM from mice fed for 6-7 months with HFD and compared them to either mice fed with a normal chow (NC) or mice fed HFD for 5-6 months followed by one month of NC, a model for relative fasting in mice^26^. We did not observe differences in the capacity to form MGCs (fig. 5A). Next, we used mice fed a HFD or subjected to intermittent fasting, i.e. 12h of HFD followed by 12h of fasting for one month (IF-HFD). Again, MGC numbers did not differ between conditions (fig. 5B). The transformation process in MGC is associated with an inflammatory response that persists until day 6^5^. We measured *Nos2* and *Il1b* mRNA expression by qPCR on day 6 of MGC transformation in cMoP from mice fed HFD vs NC for 3 months and observed persistent expression of both genes, with no significant differences between nutritional states (fig. 5C). Next, we confirmed downregulation of *Abcg1* (cholesterol efflux pump) and *Apoe*, which encodes apolipoprotein E, a cholesterol transporter that interacts with the LDL receptor^19^, but again found no significant differences between nutritional states. We conclude that metabolic alterations during HFD treatment did not alter the progenitor-inherent MGC-forming capacity.

## Discussion

A key histopathological feature in mycobacterial infections is the granuloma, which harbors different types of macrophages. This study focuses on multinucleated giant cells (MGC), which develop due to a dysregulated cell cycle following DNA damage from persistent bacterial stimuli and inflammation^5,18^. They are elite phagocytes, while offering a permissive survival niche for mycobacteria^5^. In contrast, the Langhans giant cell, another type of multinucleated macrophage found in granulomas, arises from cell fusion, it does not phagocytose efficiently but contributes to inflammation^27,28^.

Changes in lipid metabolism are closely linked to immune functions^29^. In tuberculosis (TB), lower body-mass index within a certain range is associated with increased risk of progression from latent infection to active disease^6^. Mycobacteria reprogram macrophage metabolism to promote lipid accumulation^30^, which may benefit the pathogen by creating a survival niche^31^, or, depending on the study, the host^32^. Dietary or pharmacological interventions that target lipid metabolism can shift the host–pathogen equilibrium in latent TB infection. For example, a cholesterol-rich diet has been reported to accelerate sterilization of TB lesions^10,12^. Although statin therapy improved outcomes in a murine TB model^33^, the largest randomized trial to date did not demonstrate benefit in patients^34^. Thus, defining the relationship between multinucleated giant cell (MGC) formation, one of the hallmark features of mycobacterial granulomas, and lipid metabolism may advance our understanding of this complex interaction at the cellular level. This study explored the role of LDL and oxLDL on mycobacteria-induced MGC formation. We found that both LDL and oxLDL promote MGC formation in an additive manner, suggesting distinct mechanisms. However, macrophage progenitors from mice fed a high-fat diet, which is generally associated with a higher serum level of these lipoproteins^35^, did not exhibit altered MGC forming capacities in vitro.

LDL is taken up into macrophages via a variety of pathways, thereby changing the intracellular energy store and milieu, and facilitating transformation into MGC^19^. Depletion of LDL from the macrophage milieu consequently inhibited MGC formation (fig. 2D). The requirement for LDL in transforming a macrophage is reminiscent of another type of specialized macrophage in the mycobacterial granuloma, foam cells, which are known for accumulating lipids and harboring mycobacteria during latency^36–38^. MGC exhibit some phenotypical similarities with foam cells, including lipid accumulation, altered lipid metabolism^19^, a requirement for LDL, and now an enhancement of transformative capacity by oxLDL^39^ (fig. 4). Yet, MGCs are clearly distinct from foam cells with regard to their antimicrobial capacities including enhanced phagocytosis and potent production of NO^5^. In foam cells, LDL is taken up mainly via macropinocytosis^39–41^ and after modification to oxLDL via scavenger receptors^42^. Uptake of LDL via the LDL receptor is believed to be a less relevant mechanism in foam cell formation, as LDLR is downregulated in hyperlipidemia^43^. However, LDLR-deficient macrophages showed an inherently reduced ability to form MGC (fig. 3B-C), pointing to a relevance of the LDL receptor in MGC formation.

HDL-bound cholesterol did not rescue the loss of MGC formation by delipidating FBS (fig. 2). Consequently, despite being the most prevalent lipoprotein in FBS, HDL is dispensable for the process of MGC formation. This is in accordance with the lipoprotein being generally involved in the efflux of cholesterol^44,45^, a process already reduced in MGC formation^18,19^.

The profound metabolic changes that occur with diet-induced obesity did not directly translate into altered MGC forming capacity of myeloid progenitors (fig. 5). So in addition to LDL and oxLDL modifying MGC forming capacity, changes in TB resistance in relation to body weight seem to be mediated by other mechanisms, e.g. by altered cytokine profiles^9^. Furthermore, the strong and standardized transforming stimulatory conditions applied during the in vitro differentiation of MGC may override any changes in the macrophage precursor cells induced by specific diets, thereby attenuating possible in vivo effects.

In conclusion, this study demonstrates that LDL and oxLDL are both essential and additive factors in promoting BCG-induced multinucleated giant cell (MGC) formation in vitro. In contrast, diet-induced changes in systemic lipid levels *in vivo* alone do not substantially modulate the MGC differentiation process *ex vivo*.

## Data Availability

All raw data can be shared upon request. Please contact the corresponding author.
